# Enhancing the Functional Content of Eukaryotic Protein Interaction Networks

**DOI:** 10.1371/journal.pone.0109130

**Published:** 2014-10-02

**Authors:** Gaurav Pandey, Sonali Arora, Sahil Manocha, Sean Whalen

**Affiliations:** 1 Institute for Genomics and Multiscale Biology and Department of Genetics and Genomic Sciences, Icahn School of Medicine at Mount Sinai, New York, NY, United States of America; 2 Graduate School of Biomedical Sciences, Icahn School of Medicine at Mount Sinai, New York, NY, United States of America; 3 Computational Biology, Fred Hutchinson Cancer Research Center, Seattle, WA, United States of America; 4 JP Morgan Securities Plc., London, United Kingdom; 5 Gladstone Institutes, University of California San Francisco, San Francisco, CA, United States of America; Leibniz-Institute for Farm Animal Biology (FBN), Germany

## Abstract

Protein interaction networks are a promising type of data for studying complex biological systems. However, despite the rich information embedded in these networks, these networks face important data quality challenges of noise and incompleteness that adversely affect the results obtained from their analysis. Here, we apply a robust measure of local network structure called common neighborhood similarity (CNS) to address these challenges. Although several CNS measures have been proposed in the literature, an understanding of their relative efficacies for the analysis of interaction networks has been lacking. We follow the framework of graph transformation to convert the given interaction network into a transformed network corresponding to a variety of CNS measures evaluated. The effectiveness of each measure is then estimated by comparing the quality of protein function predictions obtained from its corresponding transformed network with those from the original network. Using a large set of human and fly protein interactions, and a set of over 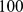 GO terms for both, we find that several of the transformed networks produce more accurate predictions than those obtained from the original network. In particular, the 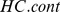 measure and other continuous CNS measures perform well this task, especially for large networks. Further investigation reveals that the two major factors contributing to this improvement are the abilities of CNS measures to prune out noisy edges and enhance functional coherence in the transformed networks.

## Introduction

Protein interaction networks are one of the most promising types of data for studying complex biological problems, such as identifying disease-related proteins and networks [1]–[3] and finding functional modules and functions of individual proteins [4], [5]. In particular, since functionally related proteins tend to be highly inter-connected in these networks, several approaches like neighborhood-based prediction [6] and FunctionalFlow [7] have been proposed for predicting the functions of unannotated proteins using this type of data [5].

However, despite the rich information embedded in protein interaction networks, they face several data quality challenges that adversely affect the results obtained from their analysis. One such pervasive problem is noise in the data, which manifests itself primarily in the form of spurious or false positive interactions [8], [9]. Studies have shown that the presence of noise in these networks has significant adverse affects on the performance of several types of analyses, including protein function prediction algorithms [10]. Another important problem facing the use of these networks is their incompleteness, i.e., the absence of biologically valid interactions from the currently available data sets [8], [9], [11]. This lack of completeness is mainly caused by the specific targeting of bait and prey proteins by individual studies (based on criteria such as functional annotations), which can only generate relatively small samples of the entire interactome of an organism. Not surprisingly, the incompleteness of such valuable data leads to missed biological insights that could be valuable [12], [13]. Thus, noise (false positives) and incompleteness (false negatives) are major challenges facing protein interaction data that need to be addressed in order to obtain richer information from them.

Here, we apply a set of techniques that make use of the local structure of an interaction network to address these challenges. For the purpose of explaining and implementing these techniques, we represent a protein interaction network as an undirected graph, with proteins being represented by nodes and interactions by edges (For this reason, the sets of terms (“network”, “graph”), (“protein”, “node”) and (“interaction”, “edge”) will be used interchangeably in this paper.). We also assume that weights reflecting the reliability of individual interactions are assigned to the corresponding edges. Most analyses of protein interaction networks are based on this representation, and focus on the direct interactions connecting two nodes.

In addition to the direct interactions, the structure of the entire protein interaction network provides information about several other types of higher-level associations between proteins. One of the most widely studied of these associations is that based on the idea of *common neighborhood* [14]–[18], where it is hypothesized that two proteins that have several common direct neighbors (interaction partners) are likely to have a *functional association* between them and vice versa. Consequently, several measures for the *common neighborhood similarity* (CNS) of two proteins, based on different variants of the number of their common neighbors, have been proposed. Several of these similarity measures have been used for clustering the proteins in the given network into functional modules [14]–[16], and many of the resultant modules were hard to discover directly from the original network. Chua *et al.* [17] used a CNS measure named FS (Functional Similarity) to predict the functions of unannotated proteins, and their approach showed better performance than several other function prediction approaches. Pandey *et al.* [18] adapted H-Confidence (HC), a measure of cohesiveness from association analysis in data mining [19], into a CNS measure for protein interaction networks.

Despite the demonstration of the utility of the different CNS measures in various contexts, an understanding of their relative efficacies for the analysis of protein interaction networks has been lacking due to several reasons. Firstly, as discussed above, each of these measures has been used for different applications involving different interaction data sets, thus making their relative comparison difficult. Furthermore, even in cases where these measures have been used in the context of function prediction [17], [18] or functional module discovery [15], [16], different sets of functional classes and evaluation measures are used, making this comparison harder. Our goal in this work is to close this gap by conducting an extensive comparative evaluation of the CNS measures within the uniform context of protein function prediction from both unweighted and weighted interaction networks. We follow the systematic framework of graph transformation [18] to generate a transformed network corresponding to each of the CNS measures evaluated. The effectiveness of each measure is then estimated by comparing the quality of function predictions made from their corresponding transformed network with those from the original network. In recent work [20], we employed this methodology for evaluating the performance of CNS measures in processing several yeast (*S. cerevisiae*) protein interaction networks. We found that CNS-based graph transformation indeed improved the quality of protein function predictions. The H-Confidence measure produced the most accurate predictions due to its ability to resist the adverse effects of noise and incompleteness in interaction data.

Proteins interaction networks of higher eukaryotes, such as human (*H. sapien*) and fly (*D. melanogaster*), are much larger, and structurally and functionally more complex than the yeast network. Furthermore, a thorough understanding of these higher networks is essential for their use in studying diseases such as cancer and cardiovascular disorders. These factors motivated us to directly investigate the efficacy of CNS measures for processing higher eukaryotic interaction networks and subsequently predicting protein function from them. Using large sets of human and fly interactions from the BioGRID database [21], and annotations with over 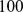 GO Biological Process terms for both, we find that several of the transformed networks produce more accurate function predictions than those obtained from the original network, although some CNS networks derived from the binary version of the original network do not perform well. Among these, the HC-based CNS measure performs well, especially for the larger human protein interaction network. Our investigation suggests that the ability of the CNS measures to identify and drop noisy edges during graph transformation is an important reason for these better predictions. CNS measures are also effective at enhancing the functional coherence, i.e., the extent to which functionally related proteins are connected by an edge, leading to more accurate function prediction than the original network. Overall, these results are expected to provide a better understanding of the efficacy of CNS measures for processing protein interaction data and the utility of these measures for enhancing the functional content of these data.

Finally, before discussing our methods and results in detail, we note that several other methods have also been proposed for assessing the reliability of protein interactions using other data sources, such as microarray data and amino acid sequences [10], [22], [23]. However, since our focus is on using the information in the given interaction network itself for this task, we do not evaluate these methods in this study. These two types of approaches provide complementary information about the reliability of an interaction, and their combination is expected to provide an even more accurate estimation of these reliabilities. However, this investigation is outside the scope of this paper.

## Materials and Methods

In this section, we discuss the interaction data set, functional annotations, CNS measures and evaluation methodology used in this study.

### Interaction data and functional annotations

We obtained our interaction data sets from the BioGRID database [21] in April, 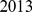. These data sets included 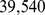 interactions between 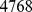 human proteins and 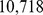 interactions between 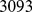 fly proteins. In addition to using the unweighted (binary) version of this network, we also generated their weighted versions, where each edge was assigned a weight equal to the fraction of the total number of studies included in the data set where it was detected.

The functional annotations for the proteins in these interaction data sets were taken from the GO database [24] in April, 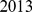. For both the organisms, we identified GO Biological Process (BP) terms assigned to at least 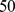 proteins included in the corresponding data sets to ensure the statistical robustness of the prediction results. Furthermore, we only selected the GO BP terms that didn't have any ancestor-descendant relationships between them. This selection reduces the effect of the hierarchical relationships between the terms, which is well-known to be a complicating factor for evaluating protein function prediction results [25]. As a result of this process, we obtained 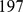 and 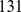 GO BP terms for human and fly respectively that were used in our evaluation and are listed in Tables S1 and S2 respectively.

### Common Neighborhood Similarity (CNS) measures

We evaluated a variety of CNS measures in our study, which are discussed below. For the purpose of defining each of these measures, we will use the following standard notation:

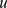
 and 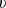 are the nodes between which the similarity is being computed.

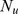
 and 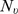 are the direct interaction partners of 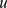 and 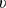 respectively, and 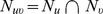.

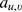
 denotes the binary or positive real-valued weight of the edge between 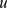 and 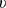.

We now define and discuss the CNS measures studied in detail.

#### Jaccard coefficient (JC)

One of the most commonly used measures for the similarity of two sets, 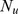 and 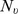 here, is the Jaccard coefficient [26], which is defined as follows: 
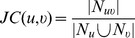
(1)


The Jaccard coefficient measures how similar the two sets are, and assumes a value of 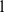 only if 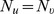. However, in this form, it can only be used for unweighted graphs. Also, this measure does not incorporate the presence or absence of an interaction between 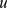 and 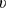 (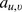) itself.

#### Pvalue (P)

Samanta *et al.* [15] proposed a probabilistic measure for the statistical significance of the common neighborhood configuration of two nodes 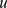 and 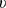 in an unweighted graph. The value of this measure, commonly known as 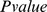 (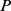), is the 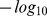 value of the probability of 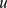 and 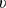 having a certain number of common neighbors by random chance, and is defined as: 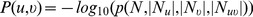
(2)


Here, 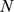 is the total number of proteins in the network, and 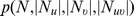 is computed on the basis of a Binomial distribution as: 
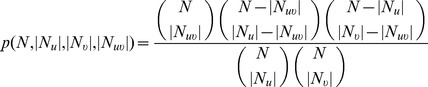
(3)


Thus, 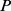 is expected to have a high value (low value of 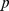) for the non-random common neighbor configurations in a network. However, similar to 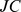, this measure is unable to take edge weights into account, thus losing information about the reliabilities of interactions over which the measure is computed. Another potential weakness of this measure is that it does not incorporate the value of 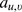, i.e., the reliability of the direct edge between 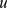 and 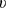.

#### Functional Similarity (FS)

Chua *et al.* [17] proposed a measure named Functional Similarity (FS) for measuring the common neighborhood similarity of two proteins in an interaction network. For an unweighted network (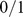 weights), this measure, referred to as 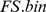, can be defined as: 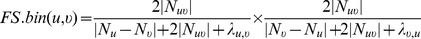
(4)where 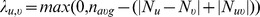 and 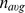 is the average number of neighbors of each protein in the network. The purpose of the 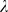 factor is to penalize the score between proteins pairs where at least one of the proteins has too few neighbors, since the score may not be very reliable in such a case. Note that unlike the other measures, the computation of 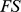 assumes that a protein, say 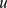, is included in its direct neighborhood, i.e., 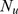.

Essentially, 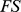 separates the (functional) similarity of two proteins into two probabilities that denote the conditional probabilities of 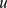 and 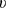 being functionally related given the neighborhoods of 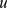 and 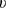 respectively. Each of these conditional probabilities are computed as how similar the set of common neighbors of 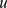 and 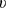 (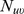) is to the set of individual neighbors of 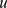 (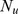) and 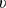 (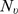). The final 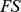 score is obtained as a product of these probabilities, assuming that they are independent.

Also, by using 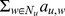 as the generalization of 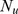 (similarly for 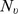), and 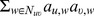 as the generalization for 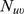, a version of the 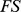 measure, named 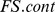, can be defined for a weighted interaction networks as follows: 
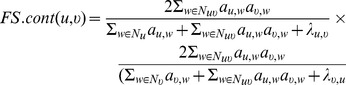
(5)


Note that we used a similar definition of 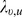 as for the unweighted network case, while using the weighted versions of 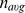, 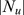, 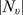 and 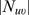. Note that Chua *et al.* [17] proposed a slightly different definition for 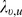 that assumes the knowledge of the functions of the proteins, which was not applicable here.

#### Topological Overlap Measure (TOM)

This measure was proposed for network analysis by Ravasz *et al.* [27] and was subsequently used for co-expression network analysis by Zhang and Horvath [16]. TOM measures the strength of the association between two nodes in a graph based on the similarity of their common neighborhood to the smaller of the individual neighborhoods of the two nodes. For the case of an unweighted or binary network, the 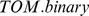 measure can be defined as: 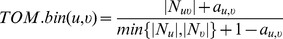
(6)


The basic definition of 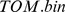 is quite straightforward. However, an important factor included in this measure is the presence or absence of an edge between 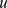 and 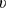 (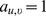 and 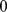 respectively) in the original network through the terms 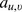 and 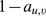 in the numerator and denominator respectively. The inclusion of these factors has the desirable effect that the value of 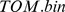 is increased if 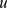 and 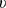 are known to have an interaction, which is sensible since the knowledge of this interaction should contribute favorably to the score for these proteins.

Again, using the same generalizations as for 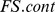 produces a formulation of 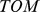 for weighted networks, i.e. 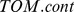, as: 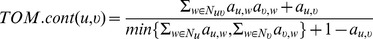
(7)


Several studies [28]–[31], have used this measure extensively for analyzing gene co-expression networks. We consider it for processing protein interactions networks.

#### H-confidence (HC)

Pandey *et al.* [18] demonstrated an innovative application of the 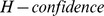 (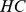) measure [19], originally designed for the analysis of binary data matrices, to the pre-processing of protein interaction networks, both weighted and unweighted. We modified the original definition of 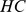 [18] slightly to define the 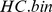 measure as: 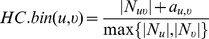
(8)


The change here is the addition of the 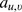 term in the numerator to incorporate the presence/absence of the interaction between 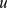 and 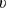. As per this definition, 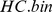 rewards cases where the set of common neighbors (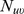) is very similar to the sets of individual neighbors of 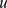 and 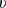. However, due to the use of the 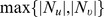 term in the numerator, 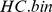 penalizes the cases where the degree of at least one of the nodes is substantially higher than 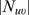, thus avoiding a bias in favor of high-degree or hub nodes in the network. This behavior of 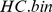 is in sharp contrast to that of the similarly defined 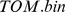 measure, the value of whose denominator is generally small for protein interaction networks due to the use of the 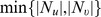 term and the fact that a vast majority of the nodes in these networks have very small degrees.

Finally, using the same generalizations as for 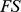 and 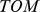, the definition of 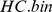 can be extended to 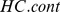 for the case of weighted interaction networks as follows: 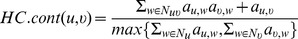
(9)


This definition of 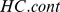 enables a more conservative estimation of 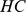-based common neighborhood similarity due to the use of the sum of the product of the edge weights, both of which are at most 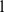 and thus their product is expected to be much smaller than the minimum of the two values. It should be noted that 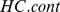 also has a behavior similar to 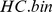, wherein nodes with low weighted degrees in the original network are more likely to have links with higher 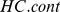 scores as compared to higher weighted degree nodes in the original network.

As can be seen, these measures adopt different formulations for computing common neighborhood similarity between two nodes (proteins) in a graph (interaction network). We next describe how we evaluated these measures within the frameworks of graph transformation and protein function prediction.

### Evaluation methodology

Our evaluation methodology consists of the following two steps:

- First, each of the above CNS measures is used to compute the similarity (strength of the association) between each pair of proteins in the input interaction network, depending on whether they operate on the weighted or unweighted version of the network. Our goal is to examine how the association networks so generated compare with the original interaction networks in terms of predicting protein function from them. However, this comparison can be biased due to varying number of edges in (size of) the networks. Thus, in the first step, we create *transformed* versions of the networks, whose number of edges is comparable to that of the original network. For this, a threshold is chosen for each CNS measure such that the number of pairs with a score higher than this threshold is as close as possible to the number of interactions in the original network. The pairs that score higher than the threshold are structured as a network, and constitute the *transformed network* for the corresponding measure. Most of our analysis is based on these transformed networks. In addition, we also examine the effect of thresholding on the results obtained after transformation.
- Next, two different protein function prediction algorithms are run on the original as well as the transformed networks to make predictions over the corresponding selected sets of GO BP process terms/classes for human and fly. The first algorithm used was Nabieva *et al.*'s FunctionalFlow algorithm [7]. We also used a simple neighborhood-based algorithm inspired by Schwikowski *et al.*'s function prediction algorithm [6]. Here, the likelihood score of a query protein performing certain function is simply counted as the sum of the weights of its interactions with proteins that are known to be annotated with that function, and these scores are collected for all the unannotated proteins in the data set for all the relevant functions. The predictions from both these algorithms are collected within a five-fold cross-validation setup.
- The collected predictions are evaluated using the 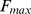 measure, which was shown to be a reliable metric in a recent large-scale protein function prediction assessment [25]. This measure is simply the maximum value of the F-measure across all the values of precision and recall at many thresholds applied to the prediction scores. To confirm the observed trends, we also evaluated the predictions using the commonly used AUC (Area under the ROC Curve) measure.

All the CNS measures and this evaluation methodology were implemented in Matlab (Mathworks). Where necessary, the computations were parallelized using Matlab's Distributed Computing toolbox. Also, note that our graph transformation process was based only on the CNS scores of the protein pairs. It is easy to introduce constraints, e.g. two proteins must be connected since they are in the same pathway, into this process by ensuring that they are satisfied in the transformed networks. However, since there is no single source of these constraints, and we didn't want to bias our results by choosing any particular source, we did not incorporate any such constraints into our methodology. We plan to study this feature in future work, and invite others as well to do the same.

## Results

In this section, we discuss the results of our evaluation, and also the subsequent analyses that we carried out to explain the observed trends.

### Details of transformed networks

Table 1 lists the details of the different transformed networks generated using the methodology described above. As can be seen, the number of interactions in these networks, as well as the number of connected proteins with at least one interaction, are almost the same as the original network, thus ensuring that the downstream analysis of these networks is not biased due to a variation in the size of the networks. The only exceptions to this observation are the 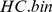 networks, which contained slightly different number of edges than the original networks, although the differences were minor. A much bigger variation is observed in the number of connected proteins, i.e. proteins with at least one edge, in the transformed networks. Here, the transformed networks derived from the binary version of the original network had substantially fewer connected proteins than the original network (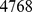 and 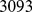 for human and fly respectively), while those derived from the continuous version had effectively the same number of connected proteins as the original network. Although this observation itself indicates a weakness of the binary CNS measures, we eliminated its influence on the rest of the evaluation by assessing the function prediction results on only the connected proteins in each network.

**Table 1:** Details of transformed networks derived from the original human and fly interaction networks using different CNS measures.

	Human			Fly		
CNS Measure	# Interactions	# Connected proteins	Range of edge weights	#Interactions	#Connected proteins	Range of edge weights
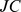	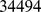	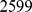	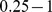	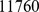	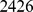	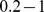
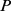	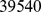	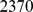	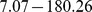	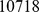	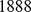	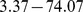
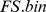	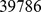	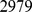	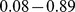	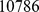	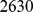	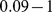
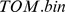	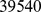	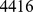	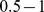	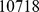	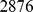	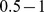
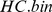	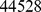	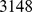	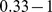	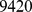	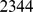	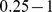
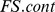	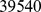	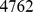	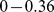	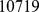	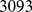	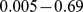
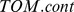	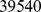	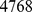	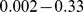	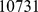	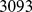	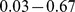
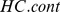	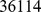	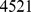	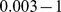	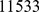	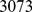	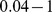

### Performance of protein function prediction algorithms

**Figure 1:**
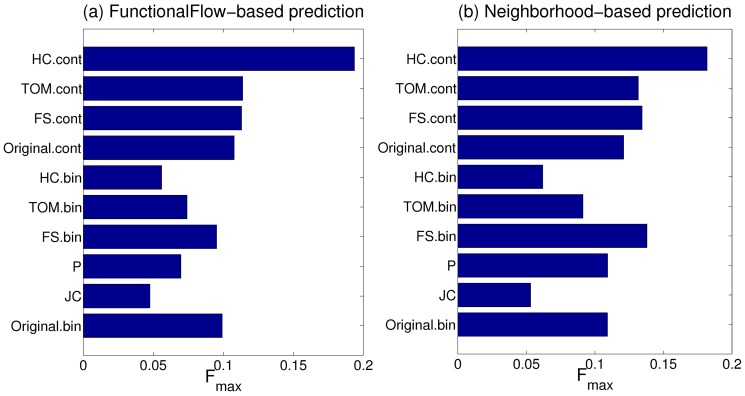
Comparison of protein function prediction results from the original and transformed human networks in terms of the median 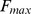 score over all the GO BP classes considered.

**Figure 2:**
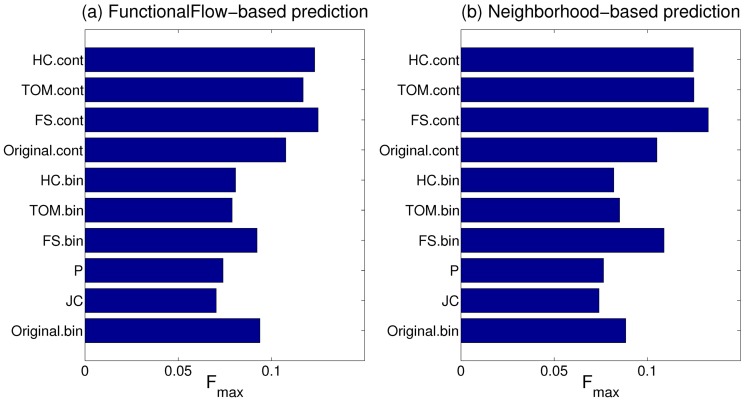
Comparison of protein function prediction results from the original and transformed fly networks in terms of the median 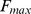 score over all the GO BP classes considered.

We evaluated the utility of each of the human and fly transformed networks for predicting the membership of their proteins in the selected GO BP classes, and compared their performance with the weighted (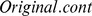) and unweighted (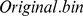) versions of the corresponding original interaction networks. Figures 1 and 2 shows this comparison for human and fly respectively in terms of the median 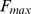 score of the FunctionalFlow and neighborhood-based function predictions over all the classes considered (Similar results based on median AUC scores are shown in Figures S1 and S2 respectively.). Table S3 lists the Wilcoxon rank sum P-values indicating the statistical significance of the improvement or deterioration of function prediction results from various CNS-transformed networks as compared to the results from the corresponding original network (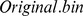 or 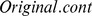). The following observations can be made from these results:

- In almost all the cases, the binary CNS measures produce much worse predictions than the 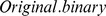 network, the only exception being 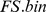, which is able to perform competitively as compared to the 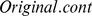 network. This inferior performance is primarily due to the loss of information incurred by these measures by not incorporating real-valued edge weights in the original network.
- In most of the cases, the continuous CNS measures that can incorporate edge weights, namely 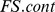, 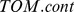 and 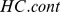, perform much better than (or almost the same as) the 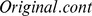 network. Furthermore, this improvement in performance is more substantial in the case of the human network as compared to the fly one. This is because of the former's larger size, from which continuous CNS measures can utilize much richer structural information to infer more reliable functional associations and thus produce more accurate function predictions.
- Among the continuous CNS measures, 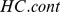 is able to make the best use of the information in the large human network to provide the largest boost in protein function prediction performance (Figure 1). For the smaller fly network, all the continuous CNS measures comparable relatively smaller improvements over the 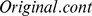 network (Figure 2), with 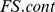 providing a slight advantage in terms of relative performance and statistical significance.

These results show that it is possible to perform more accurate analysis on the original interaction network by transforming it using appropriate CNS measures. Further, for larger interaction networks, transformation using 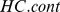 appears to provide a substantial advantage in terms of the final analysis results. Indeed, the best CNS measure for any given network needs to be determined based on rigorous evaluation, such as using our graph transformation and evaluation methodology here.

**Figure 3:**
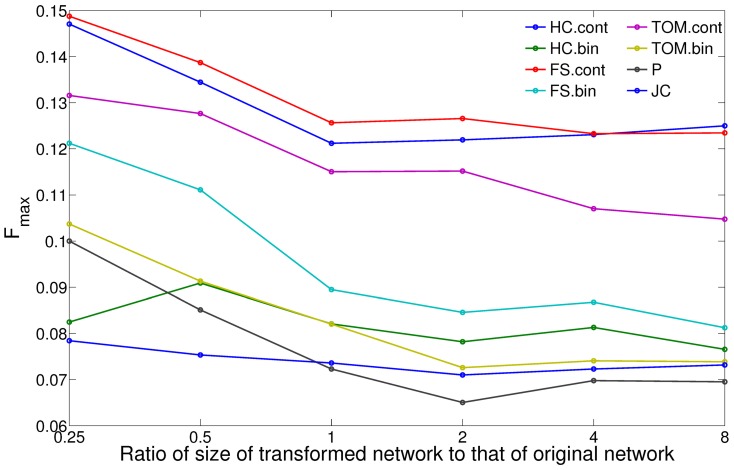
Comparison of protein function prediction results from CNS-transformed fly networks of varying sizes in terms of the median 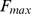 score. These transformed networks are obtained by varying the threshold on the CNS score so as to derive a transformed network with size (# edges) that is a given fraction, say 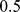, of that of the original network.

Next, we investigated how the size of the transformed networks (determined by the degree of thresholding of the respective CNS measures) influenced the quality of function predictions obtained from them. For this, using each CNS, we generated transformed networks of sizes varying from a quarter of the size of the original network to eight times the size, progressing by a factor of two in each step. Next, FunctionalFlow is run on each of these networks and the predictions evaluated as the median 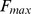 score over all the classes considered. Figure 3 shows the results of this investigation for the fly transformed networks. Interestingly, the order of performance of the CNS measures is consistent with the order in Figure 2: 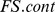 and 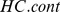 are the best performers, followed by 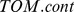, while 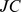 and 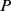 don't perform well across all thresholds. These results show that the relative efficacies of the CNS measures are not dependent on the threshold used for graph transformation. Examining the individual variation of the precision and recall scores contributing to these 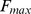 scores (Figure S3), it can be observed that this order of performance is influenced more by precision than recall. Some of the measures, such as 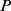, achieve high rates of recall with smaller networks, but their precision performance remains relatively low across all the sizes considered. In contrast, measures like 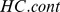 and 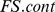 achieve balanced levels of precision and recall (approximately 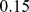
–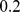). Since 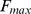 is the (conservative) harmonic mean of the two, the latter set of continuous CNS measures perform better overall as compared to binary CNS measures like 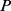 across all sizes/thresholds. Furthermore, across all these evaluation measures (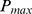, 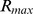 and 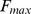), the performance of the measures becomes effectively stable when the size of the transformed network is the same as that of the original network (ratio of size = 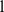) and beyond. Thus, to obtain a stable relative order of performance of these measures and the reasons underlying this performance, as well as to reduce the effect of network size on the interpretation of the results, we analyze the results at this threshold. However, users of these measures and/or our framework can use different thresholds for their analyses if they have different goals than our study, such as achieving the highest possible precision or recall.

The above results show the relative efficacies of various CNS measures when used within a graph transformation framework for problems such as protein function prediction. Now, a natural question to ask here is what features of these CNS-based transformations lend them these efficacies? We hypothesize that these features are (i) robustness of CNS measures to noise and (ii) enhancement of functional coherence after graph transformation. Through a detailed analysis of the continuous CNS measures 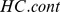, 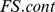 and 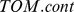, which performed the best in our function prediction experiments, we provide evidence in support of these hypotheses in the next two subsections.

### Robustness of CNS measures to noise

One of the hypotheses underlying the use of common neighborhood similarity information is that it can be used for filtering out noisy or spurious associations in a network, since two proteins connected by a true association are more likely to have a larger number of common neighbors than two proteins connected by a spurious association. We investigated if this hypothesis holds in our study, and if it contributes to better function predictions after graph transformation. Since it is difficult to identify the noisy edges in the original network a priori, we followed a simulation-based methodology for validating this hypothesis. Under this methodology, we generated several randomly perturbed versions of the 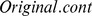 network using the random rewiring model [32], [33] where two edges in the original network are chosen randomly and two new edges are created by swapping their end points. The weights of the original edges are also randomly reassigned to the new edges. Applying this model to a varying fraction of the edges in the original network (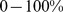) gave us several “noisy” versions of the network, and we created transformed versions of each of these networks using the continuous and binary CNS measures used in our study.

**Figure 4:**
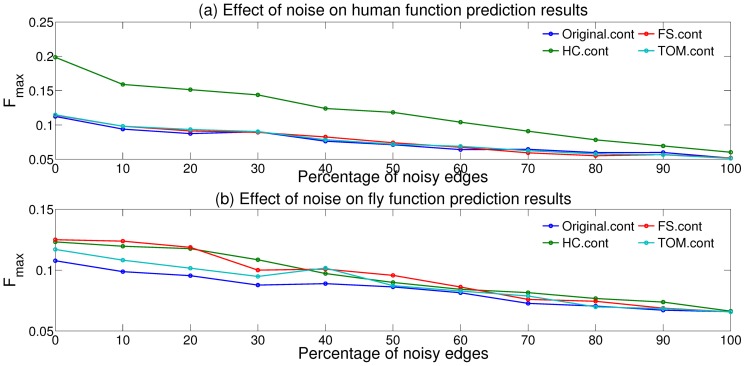
Performance of the FunctionalFlow protein function prediction algorithm, evaluated in terms the median 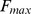 score, on the original and CNS-transformed (a) human and (b) fly networks at different levels of noise.

Next, we examined how the extent of noise in the noisy networks and their transformed versions affected the performance of the FunctionalFlow algorithm, measured in terms of the median 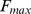 score over all the GO BP terms considered. Figure 4 shows the results of this analysis for the continuous CNS measures as the noisy fraction of the (a) human and (b) fly networks ranges from 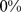 to 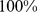. As expected, the results from all the networks, both the original and the transformed ones, become worse as the extent of noise increases, converging to a common score when the networks are completely noisy or randomized. Consistent with the order in Figure 1, 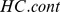 (green line) is consistently the best at resisting the effect of noise in the original human network, and can perform better than the original network even when a vast majority of the edges in the network are noisy. On the other hand, 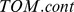 and 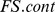 are only slightly more noise resistant than the original network, thus indicating a mechanism for how 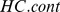 outperforms the other CNS measures in protein function prediction (Figure 1). Similarly, consistent with the overall fly function prediction results (Figure 2), 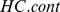 and 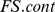 are almost equivalently noise resistant, although the relative performance from all networks is effectively the same at very high levels of noise (over 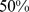). These results demonstrate the relative robustness of the continuous CNS-based transformed networks to noise in the original network, with a relative advantage to 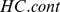 for large networks. In contrast, the same analysis for binary CNS measures (Figure S4) shows that none of these measures produce more accurate predictions than either of the original networks (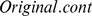 and 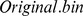) at any level of noise, except 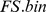 for very low levels of noise in the human network.

Overall, these results serve as validation for the hypothesis that the ability to resist the adverse effects of noise is an important factor behind the improvement of function prediction results using common neighborhood similarity quantified through continuous CNS measures.

### Enhancement of functional coherence

Three types of edges can be identified when the original network is transformed using one of the CNS measures:

- *Retained* edges: Edges in the original network that are retained after transformation due to their high CNS score.
- *Dropped* edges: Edges in the original network that are dropped due to their low CNS scores and thus are not a part of the transformed network.
- *Added* edges: Edges that were not present in the original network but were added to the transformed network due to their high CNS scores.

Given this classification, we investigated how the transformation process influences *functional coherence* (FC). Broadly, the functional coherence of a network refers to the extent to which connected proteins in the network share cellular functions and thus is inherently connected to how well algorithms like FunctionalFlow are able to predict function from this network. We define functional coherence (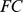) of a network 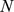, viewed as a set of edges (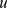,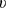), as:
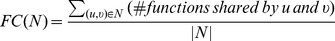
(10)


Thus, 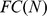 is simply the average number of functions shared by every pair of proteins connected by an edge in 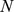.

**Figure 5:**
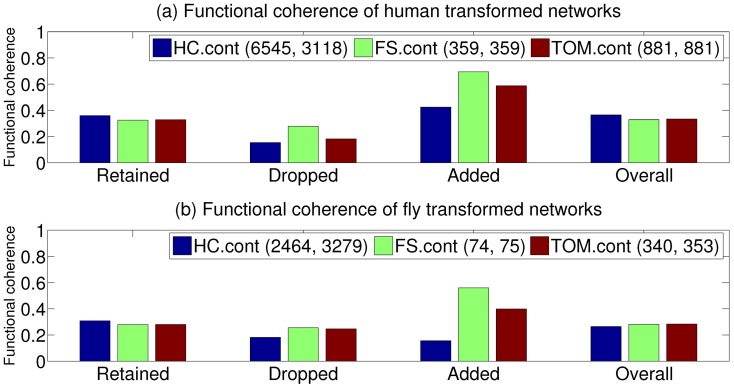
Functional relevance of the different components, namely the common, dropped and added edges, of the transformed (a) human and (b) fly networks. The legend shows the color coding of the three CNS measures examined, as well as the number of edges that were dropped from the original network and the number of edges added in their place to obtain the corresponding transformed network.

Using this definition, we evaluated the functional coherence of the above sets of edges created by *HC.cont*-, *FS.cont*- and *TOM.cont*-based transformation. Figure 5 shows the results of this evaluation for the (a) human and (b) fly networks as color-grouped bar charts. Also included in these charts are the numbers of edges dropped and added during transformation with each of these measures. The following observations can be made for both human and fly networks from these charts:

- While the number of retained edges is nearly the same for all the three measures, 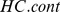 drops and adds the biggest number of edges, thus causing the biggest changes to the network structure during transformation. 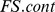 and 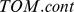 introduce minor changes to the network structure.
- The functional coherence of the edges retained by 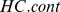 is slightly higher than 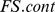 and 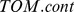, indicating that the former measure is better able to identify the most functionally coherent portion of the original network.
- The functional coherence of the edges dropped by 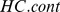 is the lowest, followed by 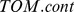 and then 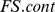, indicating that the edges dropped by 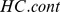 are indeed the least functionally informative and thus should not be a part of the transformed network.

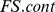
 introduces more functionally coherent edges into the transformed network, followed by 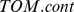 and 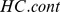.

The result of the above observations is the functional coherence of the transformed networks, whose values are shown in the last set of bars in Figure 5 (labeled “Overall”), and can be explained as follows. 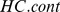 retains the most functionally coherent edges, drops the highest number of functionally incoherent edges and add a large number of reasonably functionally coherent edges. As a result, it scores the highest in terms of the overall functional coherence of its transformed network, especially for human. In contrast, although 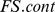 and 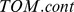 are more effective at adding functionally coherent edges, their number is fairly small, and thus this advantage is not able to counteract the disadvantages of less coherent retained edges and dropping of coherent edges. Thus, the enhanced (or deteriorated) functional coherence of the CNS-transformed network, which is inherently connected to their ability to predict protein function, serves as another factor underlying the function prediction trends discussed earlier. From this point of view, 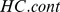 is quite effective at enhancing functional coherence, and consequently produces improvements in function prediction over the original network, which are especially substantial for the human network.

We conducted a similar analysis of how functional coherence is affected during transformation using binary CNS measures, the results of which are shown in Figure S5. These results show several contrasting trends to those in Figure 5. First, the binary measures retain very few of the edges in the original network. Second, among the large number of edges that are dropped and added during this transformation, the latter set is substantially less functionally coherent than the former. As a result, the overall functional coherence of the networks transformed using these measures is lower than those of the continuous CNS networks (Figure 5). However, among these binary measures, 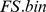 is able to attain reasonable functional coherence for both the human and fly networks. As a result, this measure is able to perform well in conjunction with the neighborhood-based function prediction algorithm (Figure 1(b)), which is based on the same principle of direct connectivity between functionally related proteins. This suggests that 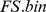 may be a good option for networks where edge weights may not be available at all.

In conclusion, we showed in this section, that while CNS-based graph transformation is generally useful, transformation based on CNS measures that are able to utilize continuous edge weights or reliabilities in the original network are especially effective for tasks such as protein function prediction. This effectiveness is due to (1) their resistance to noise in the data and (2) enhanced functional coherence of their transformed networks. In particular, 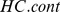 performs relatively better for large networks, such as the human one tested here, although 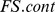 and 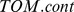 also perform well.

## Discussion

In this study, we evaluated the use of a variety of *common neighborhood similarity* (CNS) measures to quantify the association of two proteins in a protein interaction network, and used them within the graph transformation framework for processing complex eukaryotic protein interaction networks. Using a model analysis task, we showed that such processing, especially using CNS measures that take advantage of the real-valued edge reliability scores (weights), is able to substantially improve the accuracy of predictions made for several GO Biological Process terms by standard protein function prediction algorithms. In further analysis, we showed that this efficacy is achieved by boosting robustness to noise, and enhancing functional coherence to address the knowledge shortages due the incompleteness issue with currently available protein interaction data. In particular, the 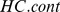 measure performs well this task, especially for large networks, due to its ability to effectively address the above challenges. Overall, the methods and results of this study should help researchers adopt robust processing schemes for protein interaction networks, which should in turn help them obtain more accurate inferences from this type of data.

We hope that this work will motivate several further research efforts. Among the most direct would be a validation of the noisy edges removed and the functional linkages added to the network during the graph transformation process using experimental PPI assessment methods, such as that of Braun *et al* [34]. Furthermore, we only explored second-degree common neighborhood-based topological features to evaluate associations between proteins. However, several other topological features, including global ones, have been studied for protein interaction networks [35]. Thus, the problem of how these features can be used within a graph transformation framework to improve analysis results should be investigated. Finally, another interesting direction would be to examine how CNS measures and other topological features perform for other types of networks that have their own characteristics, such as genetic interaction networks [36] that contain both positive and negative interactions, and regulatory and signaling networks [37] that include both directed and undirected edges.

## Supporting Information

Figure S1 Comparison of function prediction results from the original and transformed human networks in terms of the median AUC score over all the GO BP classes considered.
(TIFF)

Figure S2 Comparison of function prediction results from the original and transformed fly networks in terms of the median AUC score over all the GO BP classes considered.
(TIFF)

Figure S3Comparison of protein function prediction results from CNS-transformed fly networks of varying sizes in terms of the (a) precision (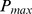) and (b) recall (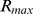) measure contributing to the median 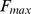 score shown in Figure 3.(TIFF)

Figure S4Performance of the FunctionalFlow protein function prediction algorithm, evaluated in terms the median 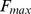 score, on the original and binary CNS-transformed (a) human and (b) fly networks at different levels of noise.(TIFF)

Figure S5 Functional relevance of the different components, namely the common, dropped and added edges, of the binary transformed (a) human and (b) fly networks. The legend shows the color coding of the five binary CNS measures examined, as well as the number of edges that were dropped from the original network and the number of edges added in their place to obtain the corresponding transformed network.
(TIFF)

Table S1 Details of selected GO Biological Process terms (classes) used for predicting the functions of human proteins in this study.
(PDF)

Table S2 Details of selected GO Biological Process terms (classes) used for predicting the functions of fly proteins in this study.
(PDF)

Table S3Wilcoxon rank sum P-values indicating the statistical significance of the improvement or deterioration of function prediction results from various CNS-transformed networks as compared to the results from the corresponding original network (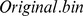 or 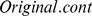).(PDF)
