# Supplementary figures and images for: Enhancing the Functional Content of Eukaryotic Protein Interaction Networks
Source: PLoS One. 2014 Oct 2;9(10):e109130. doi: 10.1371/journal.pone.0109130 (PMC4183583; doi:10.1371/journal.pone.0109130)

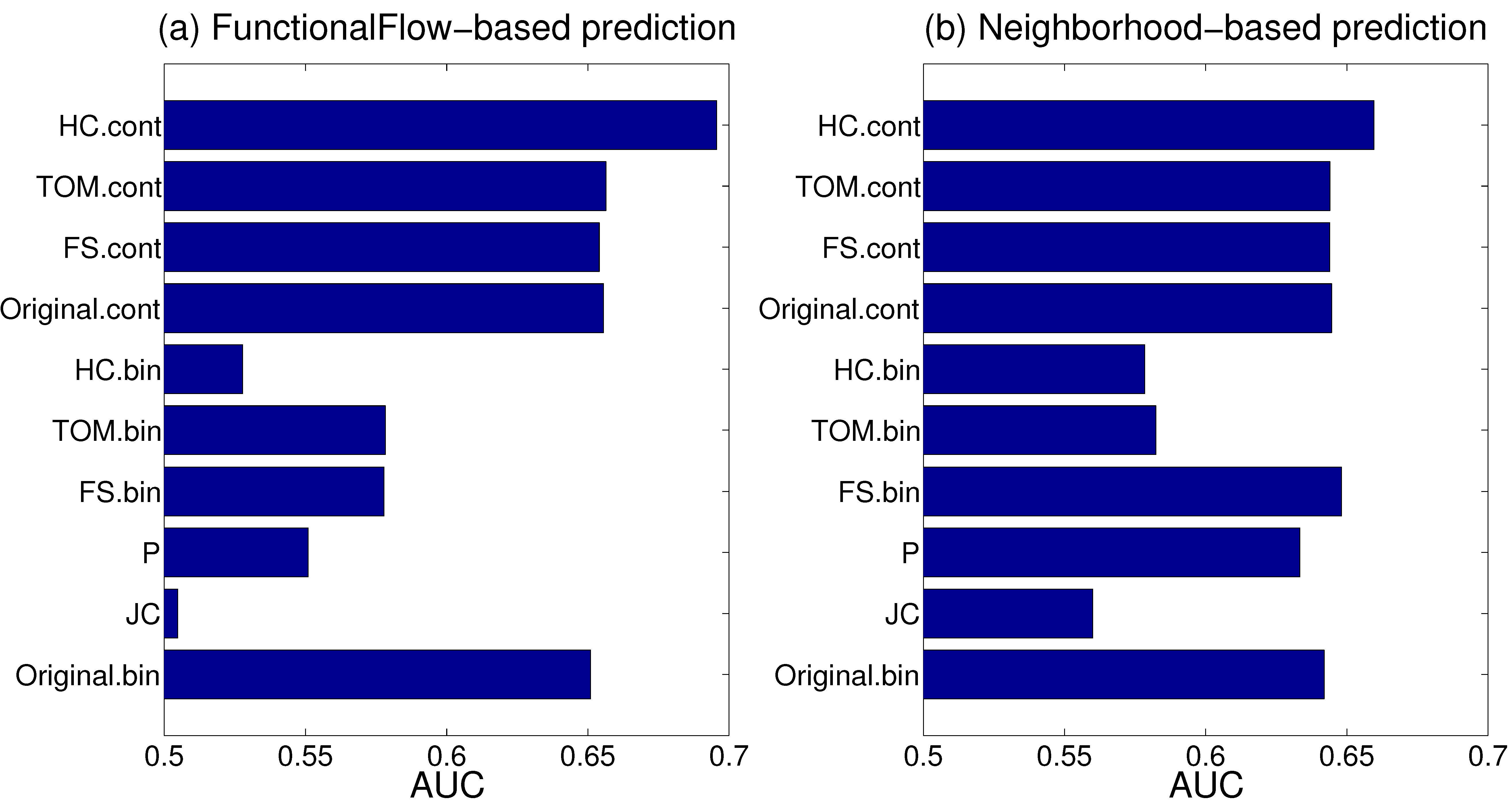

Supplement: Figure S1 — Comparison of function prediction results from the original and transformed human networks in terms of the median AUC score over all the GO BP classes considered. (TIFF) [file pone.0109130.s001.tiff]

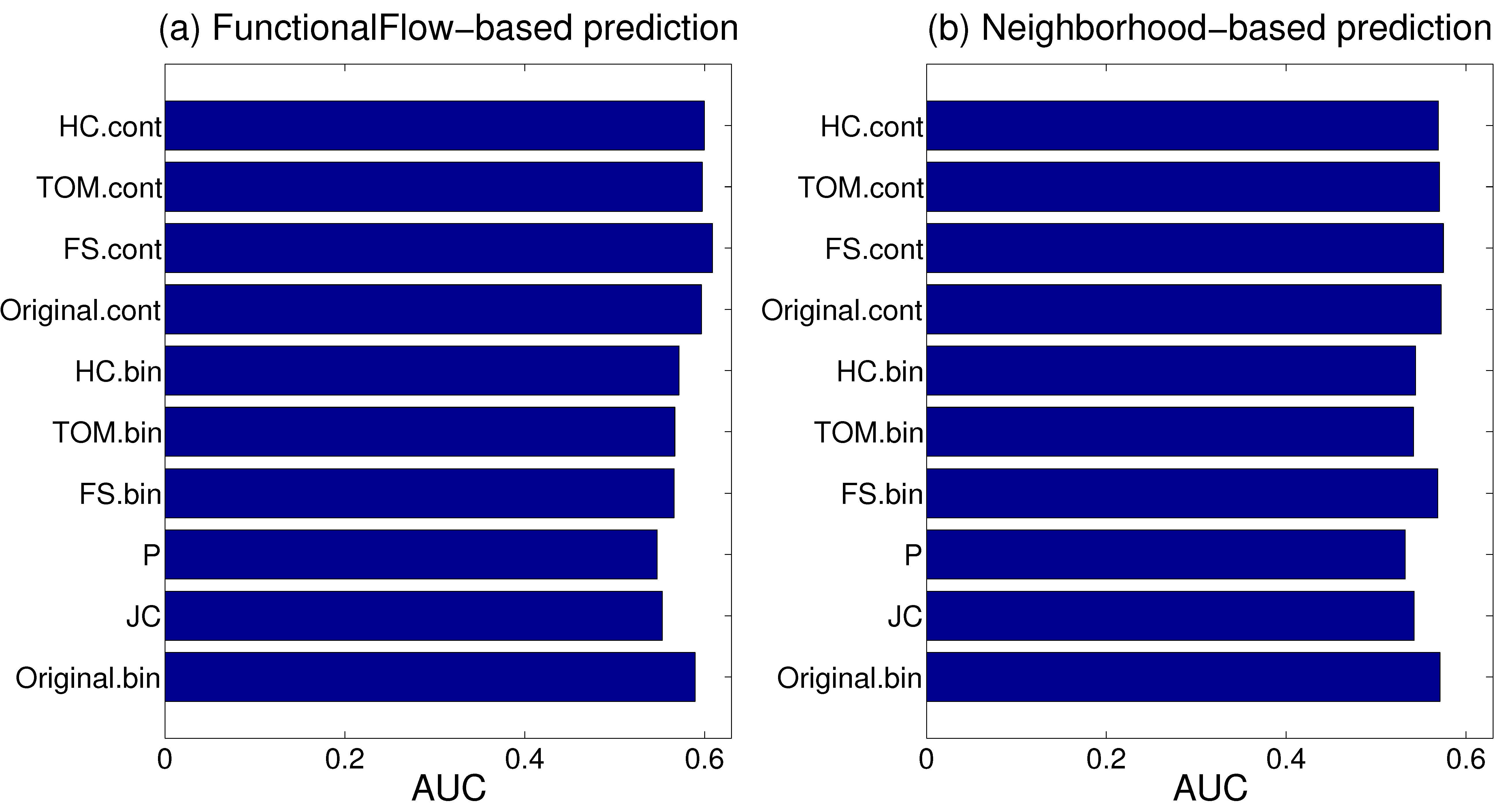

Supplement: Figure S2 — Comparison of function prediction results from the original and transformed fly networks in terms of the median AUC score over all the GO BP classes considered. (TIFF) [file pone.0109130.s002.tiff]

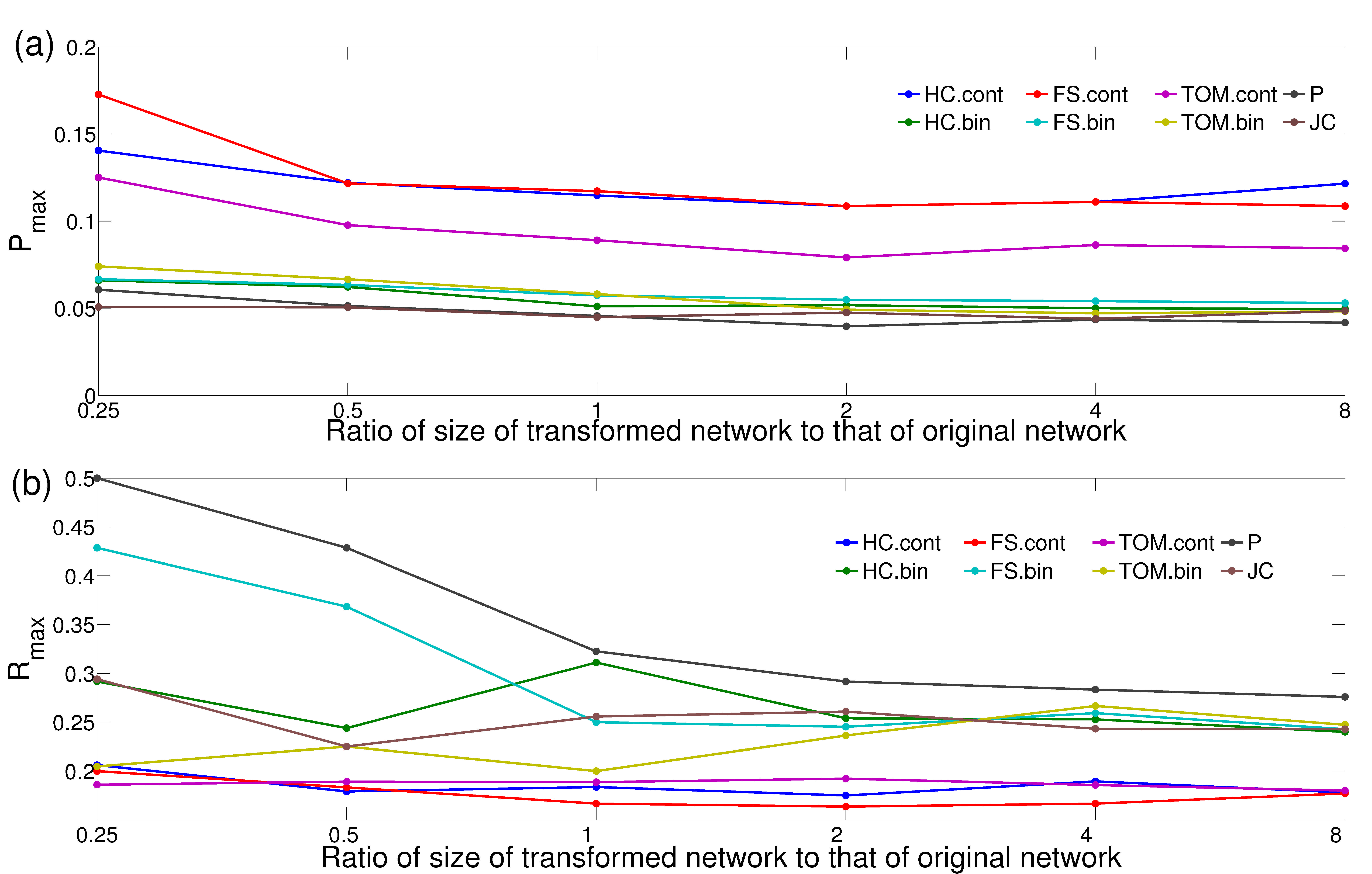

Supplement: Figure S3 — Comparison of protein function prediction results from CNS-transformed fly networks of varying sizes in terms of the (a) precision () and (b) recall () measure contributing to the median score shown in Figure 3. (TIFF) [file pone.0109130.s003.tiff]

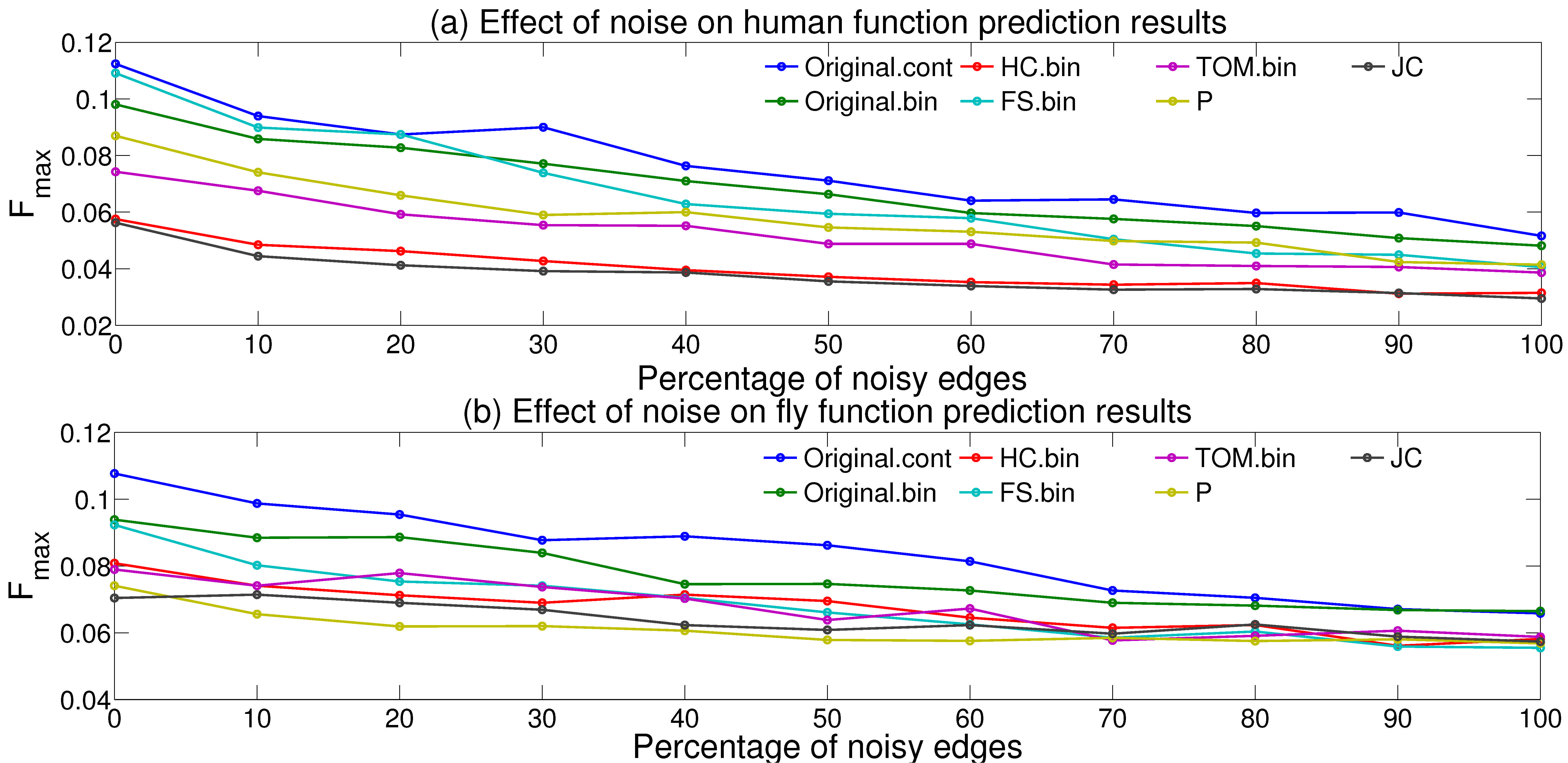

Supplement: Figure S4 — Performance of the FunctionalFlow protein function prediction algorithm, evaluated in terms the median score, on the original and binary CNS-transformed (a) human and (b) fly networks at different levels of noise. (TIFF) [file pone.0109130.s004.tiff]

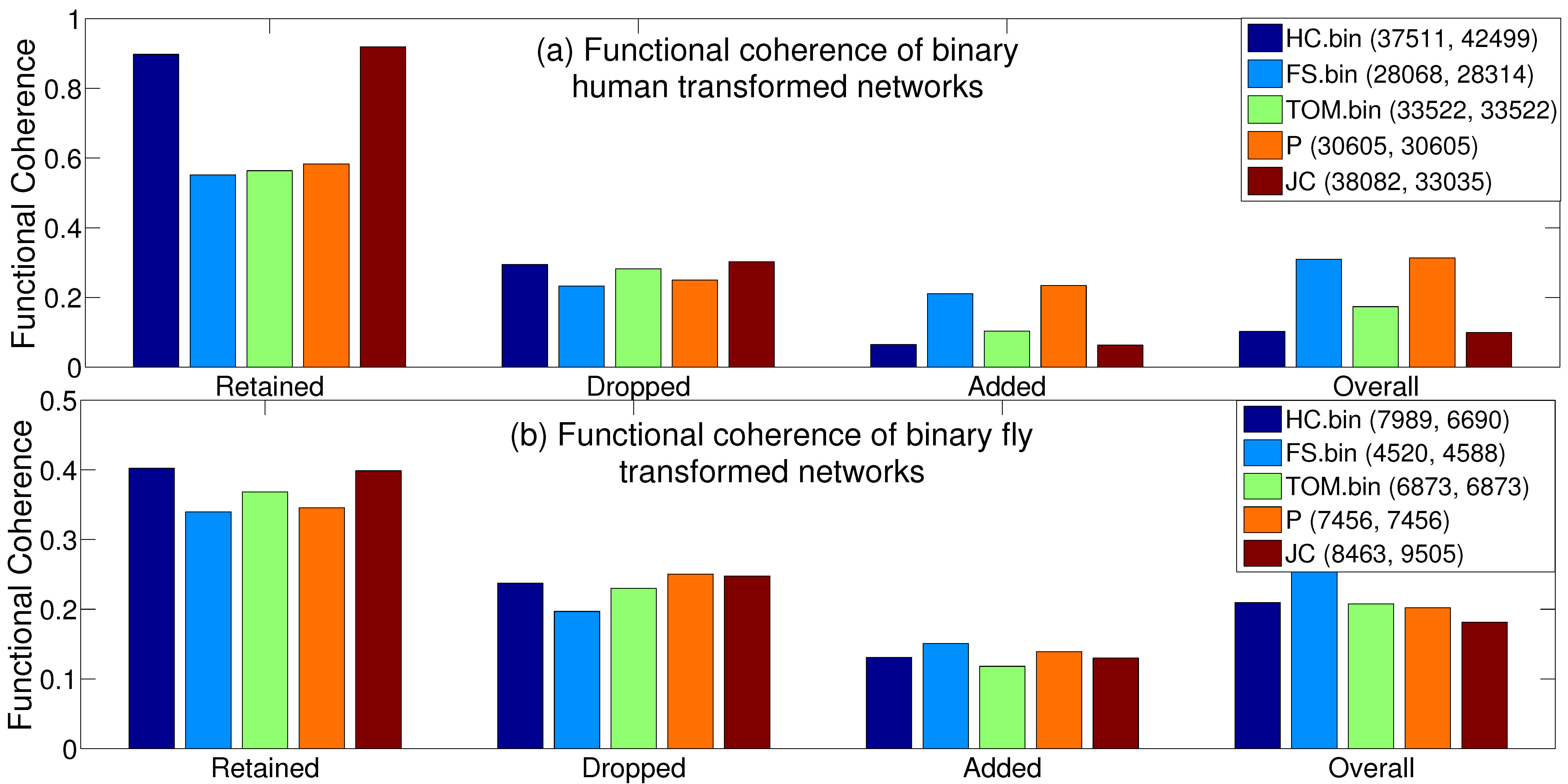

Supplement: Figure S5 — Functional relevance of the different components, namely the common, dropped and added edges, of the binary transformed (a) human and (b) fly networks. The legend shows the color coding of the five binary CNS measures examined, as well as the number of edges that were dropped from the original network and the number of edges added in their place to obtain the corresponding transformed network. (TIFF) [file pone.0109130.s005.tiff]
