# Supplementary material for: Enhancing the Functional Content of Eukaryotic Protein Interaction Networks
Source: PLoS One. 2014 Oct 2;9(10):e109130. doi: 10.1371/journal.pone.0109130 (PMC4183583; doi:10.1371/journal.pone.0109130)
Supplement: Table S1 — Details of selected GO Biological Process terms (classes) used for predicting the functions of human proteins in this study. (PDF) [file pone.0109130.s006.pdf]

GO Term ID	Definition	Size
GO:0045944	positive regulation of transcription from RNA polymerase II promoter	329
GO:0008284	positive regulation of cell proliferation	225
GO:0000122	negative regulation of transcription from RNA polymerase II promoter	212
GO:0043066	negative regulation of apoptotic process	212
GO:0008285	negative regulation of cell proliferation	183
GO:0016482	cytoplasmic transport	179
GO:1901701	cellular response to oxygen-containing compound	168
GO:0045597	positive regulation of cell differentiation	158
GO:0043065	positive regulation of apoptotic process	136
GO:0048468	cell development	127
GO:0030335	positive regulation of cell migration	125
GO:0045596	negative regulation of cell differentiation	125
GO:0010638	positive regulation of organelle organization	118
GO:0016044	cellular membrane organization	113
GO:0000398	mRNA splicing, via spliceosome	110
GO:0000226	microtubule cytoskeleton organization	106
GO:0071363	cellular response to growth factor stimulus	105
GO:0007169	transmembrane receptor protein tyrosine kinase signaling pathway	102
GO:0043123	positive regulation of I-kappaB kinase/NF-kappaB cascade	102
GO:0046777	protein autophosphorylation	101
GO:1901566	organonitrogen compound biosynthetic process	100
GO:0055114	oxidation-reduction process	97
GO:0000209	protein polyubiquitination	96
GO:0051259	protein oligomerization	94
GO:0007005	mitochondrion organization	92
GO:0009887	organ morphogenesis	91
GO:0045786	negative regulation of cell cycle	90
GO:0050678	regulation of epithelial cell proliferation	90
GO:0051098	regulation of binding	89
GO:0001819	positive regulation of cytokine production	87
GO:0009154	purine ribonucleotide catabolic process	87
GO:0070925	organelle assembly	87
GO:1901137	carbohydrate derivative biosynthetic process	87
GO:0006366	transcription from RNA polymerase II promoter	86
GO:0010639	negative regulation of organelle organization	86
GO:0000902	cell morphogenesis	84
GO:0006469	negative regulation of protein kinase activity	84
GO:0071214	cellular response to abiotic stimulus	84
GO:0009611	response to wounding	83
GO:0030036	actin cytoskeleton organization	83
GO:0043161	proteasomal ubiquitin-dependent protein catabolic process	83
GO:0046130	purine ribonucleoside catabolic process	83
GO:0006605	protein targeting	82
GO:0019216	regulation of lipid metabolic process	82
GO:0043623	cellular protein complex assembly	82
GO:0044057	regulation of system process	81
GO:0007059	chromosome segregation	80
GO:0008610	lipid biosynthetic process	80
GO:0009615	response to virus	79
GO:0022613	ribonucleoprotein complex biogenesis	79

GO:0030030	cell projection organization	79
GO:0030097	hemopoiesis	79
GO:2001020	regulation of response to DNA damage stimulus	79
GO:0001666	response to hypoxia	78
GO:0006520	cellular amino acid metabolic process	78
GO:0006897	endocytosis	78
GO:0016197	endosomal transport	78
GO:0009207	purine ribonucleoside triphosphate catabolic process	76
GO:0043433	negative regulation of sequence-specific DNA binding transcription factor activity	76
GO:0071496	cellular response to external stimulus	76
GO:0006417	regulation of translation	75
GO:0010741	negative regulation of intracellular protein kinase cascade	75
GO:0045765	regulation of angiogenesis	75
GO:0071417	cellular response to organonitrogen compound	75
GO:0007507	heart development	74
GO:0008283	cell proliferation	74
GO:0031331	positive regulation of cellular catabolic process	74
GO:0031396	regulation of protein ubiquitination	74
GO:0045637	regulation of myeloid cell differentiation	74
GO:0007186	G-protein coupled receptor signaling pathway	73
GO:0033044	regulation of chromosome organization	73
GO:0060828	regulation of canonical Wnt receptor signaling pathway	73
GO:0006412	translation	72
GO:0006913	nucleocytoplasmic transport	72
GO:0031344	regulation of cell projection organization	72
GO:0071396	cellular response to lipid	72
GO:0090068	positive regulation of cell cycle process	72
GO:0032870	cellular response to hormone stimulus	71
GO:0060627	regulation of vesicle-mediated transport	71
GO:0032147	activation of protein kinase activity	70
GO:0050877	neurological system process	70
GO:0044723	single-organism carbohydrate metabolic process	69
GO:0016573	histone acetylation	68
GO:0022414	reproductive process	68
GO:0032956	regulation of actin cytoskeleton organization	68
GO:0044283	small molecule biosynthetic process	68
GO:2001234	negative regulation of apoptotic signaling pathway	68
GO:0030336	negative regulation of cell migration	67
GO:0034470	ncRNA processing	67
GO:0050731	positive regulation of peptidyl-tyrosine phosphorylation	67
GO:0016337	cell-cell adhesion	66
GO:0031647	regulation of protein stability	66
GO:0032103	positive regulation of response to external stimulus	66
GO:0042306	regulation of protein import into nucleus	66
GO:0046328	regulation of JNK cascade	66
GO:0006874	cellular calcium ion homeostasis	65
GO:0030308	negative regulation of cell growth	65
GO:0071407	cellular response to organic cyclic compound	65
GO:0071705	nitrogen compound transport	65
GO:0090092	regulation of transmembrane receptor protein serine/threonine kinase signaling pathway	65

GO:0002697	regulation of immune effector process	64
GO:0006302	double-strand break repair	64
GO:0034765	regulation of ion transmembrane transport	64
GO:0051092	positive regulation of NF-kappaB transcription factor activity	64
GO:2001236	regulation of extrinsic apoptotic signaling pathway	64
GO:0030855	epithelial cell differentiation	63
GO:0007067	mitosis	62
GO:0051047	positive regulation of secretion	62
GO:2000021	regulation of ion homeostasis	62
GO:0006631	fatty acid metabolic process	61
GO:0043280	positive regulation of cysteine-type endopeptidase activity involved in apoptotic process	61
GO:0043414	macromolecule methylation	60
GO:0044403	symbiosis, encompassing mutualism through parasitism	60
GO:0045787	positive regulation of cell cycle	60
GO:0010921	regulation of phosphatase activity	59
GO:0016579	protein deubiquitination	59
GO:0042493	response to drug	59
GO:0061061	muscle structure development	59
GO:0008015	blood circulation	58
GO:0010769	regulation of cell morphogenesis involved in differentiation	58
GO:0044282	small molecule catabolic process	58
GO:0051251	positive regulation of lymphocyte activation	58
GO:0097193	intrinsic apoptotic signaling pathway	58
GO:1901991	negative regulation of mitotic cell cycle phase transition	58
GO:0000077	DNA damage checkpoint	57
GO:0009411	response to UV	57
GO:0032886	regulation of microtubule-based process	57
GO:0042391	regulation of membrane potential	57
GO:0043406	positive regulation of MAP kinase activity	57
GO:0045321	leukocyte activation	57
GO:0045664	regulation of neuron differentiation	57
GO:0050792	regulation of viral process	57
GO:0051924	regulation of calcium ion transport	57
GO:0001818	negative regulation of cytokine production	56
GO:0010038	response to metal ion	56
GO:0010594	regulation of endothelial cell migration	56
GO:0019221	cytokine-mediated signaling pathway	56
GO:0022403	cell cycle phase	56
GO:0034599	cellular response to oxidative stress	56
GO:0035023	regulation of Rho protein signal transduction	56
GO:0050863	regulation of T cell activation	56
GO:0071103	DNA conformation change	56
GO:0090287	regulation of cellular response to growth factor stimulus	56
GO:0000910	cytokinesis	55
GO:0002683	negative regulation of immune system process	55
GO:0006470	protein dephosphorylation	55
GO:0007267	cell-cell signaling	55
GO:0010212	response to ionizing radiation	55
GO:0022411	cellular component disassembly	55
GO:0032409	regulation of transporter activity	55
GO:0034220	ion transmembrane transport	55

GO:0048729	tissue morphogenesis	55
GO:0050920	regulation of chemotaxis	55
GO:0060326	cell chemotaxis	55
GO:2001242	regulation of intrinsic apoptotic signaling pathway	55
GO:0006275	regulation of DNA replication	54
GO:0010951	negative regulation of endopeptidase activity	54
GO:0030278	regulation of ossification	54
GO:0045785	positive regulation of cell adhesion	54
GO:0000165	MAPK cascade	53
GO:0002253	activation of immune response	53
GO:0006820	anion transport	53
GO:0007417	central nervous system development	53
GO:0009790	embryo development	53
GO:0030178	negative regulation of Wnt receptor signaling pathway	53
GO:0050708	regulation of protein secretion	53
GO:0051054	positive regulation of DNA metabolic process	53
GO:0051224	negative regulation of protein transport	53
GO:0051640	organelle localization	53
GO:0090066	regulation of anatomical structure size	53
GO:0090316	positive regulation of intracellular protein transport	53
GO:0002252	immune effector process	52
GO:0006066	alcohol metabolic process	52
GO:0018209	peptidyl-serine modification	52
GO:0032320	positive regulation of Ras GTPase activity	52
GO:0032496	response to lipopolysaccharide	52
GO:0045732	positive regulation of protein catabolic process	52
GO:0045862	positive regulation of proteolysis	52
GO:0046034	ATP metabolic process	52
GO:0046903	secretion	52
GO:0051186	cofactor metabolic process	52
GO:0070838	divalent metal ion transport	52
GO:0007178	transmembrane receptor protein serine/threonine kinase signaling pathway	51
GO:0009165	nucleotide biosynthetic process	51
GO:0031334	positive regulation of protein complex assembly	51
GO:0045087	innate immune response	51
GO:0097202	activation of cysteine-type endopeptidase activity	51
GO:0001525	angiogenesis	50
GO:0006402	mRNA catabolic process	50
GO:0030182	neuron differentiation	50
GO:0034504	protein localization to nucleus	50
GO:0040029	regulation of gene expression, epigenetic	50
GO:0044772	mitotic cell cycle phase transition	50
GO:0045088	regulation of innate immune response	50
GO:0061136	regulation of proteasomal protein catabolic process	50
GO:0070374	positive regulation of ERK1 and ERK2 cascade	50
GO:0097194	execution phase of apoptosis	50
