# Supplementary material for: Enhancing the Functional Content of Eukaryotic Protein Interaction Networks
Source: PLoS One. 2014 Oct 2;9(10):e109130. doi: 10.1371/journal.pone.0109130 (PMC4183583; doi:10.1371/journal.pone.0109130)
Supplement: Table S2 — Details of selected GO Biological Process terms (classes) used for predicting the functions of fly proteins in this study. (PDF) [file pone.0109130.s007.pdf]

GO Term ID	Definition	Size
GO:0007476	imaginal disc-derived wing morphogenesis	237
GO:0007411	axon guidance	152
GO:0046331	lateral inhibition	144
GO:0006909	phagocytosis	142
GO:0045944	positive regulation of transcription from RNA polymerase II promoter	137
GO:0007163	establishment or maintenance of cell polarity	133
GO:0000398	mRNA splicing, via spliceosome	132
GO:0006259	DNA metabolic process	126
GO:0008283	cell proliferation	120
GO:0034645	cellular macromolecule biosynthetic process	120
GO:0044087	regulation of cellular component biogenesis	117
GO:0009968	negative regulation of signal transduction	115
GO:0048813	dendrite morphogenesis	113
GO:0007067	mitosis	111
GO:0042127	regulation of cell proliferation	102
GO:0009967	positive regulation of signal transduction	96
GO:0010608	posttranscriptional regulation of gene expression	96
GO:0007059	chromosome segregation	95
GO:0008340	determination of adult lifespan	95
GO:0048589	developmental growth	95
GO:0006955	immune response	94
GO:0007626	locomotory behavior	93
GO:0070925	organelle assembly	93
GO:0010927	cellular component assembly involved in morphogenesis	92
GO:0009953	dorsal/ventral pattern formation	89
GO:0007420	brain development	88
GO:0002682	regulation of immune system process	84
GO:0007155	cell adhesion	84
GO:0008360	regulation of cell shape	84
GO:0051656	establishment of organelle localization	84
GO:0001751	compound eye photoreceptor cell differentiation	83
GO:0007298	border follicle cell migration	82
GO:0007015	actin filament organization	80
GO:0042981	regulation of apoptotic process	79
GO:0034613	cellular protein localization	78
GO:0040029	regulation of gene expression, epigenetic	78
GO:0051049	regulation of transport	78
GO:0060341	regulation of cellular localization	77
GO:0051705	multi-organism behavior	76
GO:0001709	cell fate determination	75
GO:0044281	small molecule metabolic process	75
GO:0048638	regulation of developmental growth	75
GO:0000022	mitotic spindle elongation	73
GO:0030534	adult behavior	73
GO:0006403	RNA localization	72
GO:0007519	skeletal muscle tissue development	72
GO:0045664	regulation of neuron differentiation	72
GO:0051298	centrosome duplication	72
GO:1901564	organonitrogen compound metabolic process	72
GO:0000122	negative regulation of transcription from RNA polymerase II promoter	71

GO:0051130	positive regulation of cellular component organization	71
GO:0000910	cytokinesis	70
GO:0002164	larval development	70
GO:0007167	enzyme linked receptor protein signaling pathway	70
GO:0007286	spermatid development	70
GO:0009416	response to light stimulus	70
GO:0015031	protein transport	70
GO:0022416	chaeta development	70
GO:0032535	regulation of cellular component size	70
GO:0050808	synapse organization	70
GO:0051129	negative regulation of cellular component organization	70
GO:0031399	regulation of protein modification process	69
GO:0044265	cellular macromolecule catabolic process	69
GO:0007391	dorsal closure	68
GO:0010627	regulation of intracellular protein kinase cascade	68
GO:0060249	anatomical structure homeostasis	68
GO:0060562	epithelial tube morphogenesis	68
GO:0016570	histone modification	67
GO:0034654	nucleobase-containing compound biosynthetic process	67
GO:0042742	defense response to bacterium	67
GO:0007447	imaginal disc pattern formation	66
GO:0050790	regulation of catalytic activity	66
GO:0007349	cellularization	65
GO:0019098	reproductive behavior	65
GO:0007304	chorion-containing eggshell formation	64
GO:0051094	positive regulation of developmental process	64
GO:0007293	germarium-derived egg chamber formation	63
GO:0009266	response to temperature stimulus	63
GO:0016458	gene silencing	63
GO:0035556	intracellular signal transduction	63
GO:0007095	mitotic G2 DNA damage checkpoint	61
GO:0007600	sensory perception	61
GO:0016482	cytoplasmic transport	61
GO:0042048	olfactory behavior	61
GO:0008038	neuron recognition	60
GO:0019220	regulation of phosphate metabolic process	60
GO:0051493	regulation of cytoskeleton organization	60
GO:0001736	establishment of planar polarity	59
GO:0031344	regulation of cell projection organization	59
GO:0097305	response to alcohol	59
GO:0006468	protein phosphorylation	57
GO:0007507	heart development	57
GO:0006508	proteolysis	56
GO:0007480	imaginal disc-derived leg morphogenesis	56
GO:0042461	photoreceptor cell development	56
GO:0048747	muscle fiber development	56
GO:0050807	regulation of synapse organization	56
GO:0007613	memory	55
GO:0008593	regulation of Notch signaling pathway	55
GO:0043900	regulation of multi-organism process	55
GO:0071310	cellular response to organic substance	55
GO:0006461	protein complex assembly	54

GO:0007618	mating	54
GO:0017145	stem cell division	54
GO:0019827	stem cell maintenance	54
GO:0042335	cuticle development	54
GO:0072002	Malpighian tubule development	54
GO:0006629	lipid metabolic process	53
GO:0007143	female meiosis	53
GO:0007498	mesoderm development	53
GO:0035071	salivary gland cell autophagic cell death	53
GO:0043484	regulation of RNA splicing	53
GO:0048608	reproductive structure development	53
GO:0080135	regulation of cellular response to stress	53
GO:0016202	regulation of striated muscle tissue development	52
GO:0031329	regulation of cellular catabolic process	52
GO:0035152	regulation of tube architecture, open tracheal system	52
GO:0044706	multi-multicellular organism process	52
GO:2000027	regulation of organ morphogenesis	52
GO:0007623	circadian rhythm	51
GO:0045137	development of primary sexual characteristics	51
GO:0048546	digestive tract morphogenesis	51
GO:0050684	regulation of mRNA processing	51
GO:0006915	apoptotic process	50
GO:0007314	oocyte anterior/posterior axis specification	50
GO:0008544	epidermis development	50
GO:0019725	cellular homeostasis	50
GO:0044093	positive regulation of molecular function	50
GO:0045926	negative regulation of growth	50
GO:0045995	regulation of embryonic development	50
GO:0051247	positive regulation of protein metabolic process	50
