# Supplementary material for: Enhancing the Functional Content of Eukaryotic Protein Interaction Networks
Source: PLoS One. 2014 Oct 2;9(10):e109130. doi: 10.1371/journal.pone.0109130 (PMC4183583; doi:10.1371/journal.pone.0109130)
Supplement: Table S3 — Wilcoxon rank sum P-values indicating the statistical significance of the improvement or deterioration of function prediction results from various CNS-transformed networks as compared to the results from the corresponding original network ( or ). (PDF) [file pone.0109130.s008.pdf]

	Human		Fly	
CNS Measure	FunctionalFlow	Neighborhood	FunctionalFlow	Neighborhood
JC	2.2060×10^{-21}	5.0304×10^{-21}	1.4415×10^{-6}	0.0039
P	0.0495	0.9161	3.3036×10^{-5}	0.0533
$FS.bin$	0.2039	8.5403×10^{-4}	0.2186	0.0378
$TOM.bin$	2.7304×10^{-6}	0.0018	0.0078	0.5420
$HC.bin$	7.6277×10^{-20}	7.9610×10^{-15}	0.0063	0.2626
$FS.cont$	0.8972	0.0102	0.0115	4.0388×10^{-4}
$TOM.cont$	0.5667	0.1148	0.3835	0.0097
$HC.cont$	1.7239×10^{-21}	4.9478×10^{-13}	0.1786	0.0054
